# The Role of Methemoglobin and Carboxyhemoglobin in COVID-19: A Review

**DOI:** 10.3390/jcm10010050

**Published:** 2020-12-25

**Authors:** Felix Scholkmann, Tanja Restin, Marco Ferrari, Valentina Quaresima

**Affiliations:** 1 Biomedical Optics Research Laboratory, Department of Neonatology, University Hospital Zurich, University of Zurich, 8091 Zurich, Switzerland; 2 Newborn Research Zurich, Department of Neonatology, University Hospital Zurich, University of Zurich, 8091 Zurich, Switzerland; Tanja.Restin@usz.ch; 3 Department of Life, Health and Environmental Sciences, University of L’Aquila, 67100 L’Aquila, Italy; marco.ferrari@univaq.it (M.F.); valentina.quaresima@univaq.it (V.Q.)

**Keywords:** COVID-19, SARS-CoV-2, methemoglobin, methemoglobinemia, carboxyhemoglobin, carboxyhemoglobinemia

## Abstract

Following the outbreak of a novel coronavirus (SARS-CoV-2) associated with pneumonia in China (Corona Virus Disease 2019, COVID-19) at the end of 2019, the world is currently facing a global pandemic of infections with SARS-CoV-2 and cases of COVID-19. Since severely ill patients often show elevated methemoglobin (MetHb) and carboxyhemoglobin (COHb) concentrations in their blood as a marker of disease severity, we aimed to summarize the currently available published study results (case reports and cross-sectional studies) on MetHb and COHb concentrations in the blood of COVID-19 patients. To this end, a systematic literature research was performed. For the case of MetHb, seven publications were identified (five case reports and two cross-sectional studies), and for the case of COHb, three studies were found (two cross-sectional studies and one case report). The findings reported in the publications show that an increase in MetHb and COHb can happen in COVID-19 patients, especially in critically ill ones, and that MetHb and COHb can increase to dangerously high levels during the course of the disease in some patients. The medications given to the patient and the patient’s glucose-6-phospate dehydrogenase (G6PD) status seem to be important factors determining the severity of the methemoglobinemia and carboxyhemoglobinemia. Therefore, G6PD status should be determined before medications such as hydroxychloroquine are administered. In conclusion, MetHb and COHb can be elevated in COVID-19 patients and should be checked routinely in order to provide adequate medical treatment as well as to avoid misinterpretation of fingertip pulse oximetry readings, which can be inaccurate and unreliable in case of elevated MetHb and COHb levels in the blood.

## 1. Introduction

After a series of acute respiratory syndrome cases of unknown cause were reported in December 2019 in Wuhan, Hubei province, China, genome sequencing of lower respiratory tract samples from patients showed the presence of a novel human coronavirus (severe acute respiratory syndrome coronavirus 2, SARS-CoV-2) [1,2] and the disease associated with SARS-CoV-2 was termed Corona Virus Disease 2019 (COVID-19). On 11 March 2020, the World Health Organization (WHO) declared the outbreak a global pandemic. Until now (November 2020), more than 47 million COVID-19 cases have been reported globally by the WHO.

While most of SARS-CoV-2 infected subjects are pauci-symptomatic [3] and about 15% asymptomatic [4], in some individuals the disease can develop to a more serious respiratory illness requiring hospitalization and intensive care treatment when it progresses to a critical illness involving pneumonia with hypoxemia, acute respiratory distress syndrome and severe systemic inflammation [5]. The infection-fatality rate (IFR) is determined currently to be 0.68% (0.53–0.82%) [6], with a decrease in the number to be expected due to the current increased divergence between the infection and death rates globally. The IFR is a function of age, with an IFR of <0.01% for subjects under 25 years of age and a log-linear increase of the IFR for subjects older than 30 years [7].

As critically ill patients are often found to form methemoglobin (MetHb) [8,9,10] and carboxyhemoglobin (COHb) [11,12], MetHb and COHb formation might also be relevant in case of COVID-19. MetHb is formed when the ferrous iron (Fe^2+^) of the heme group of hemoglobin (Hb) is oxidized to ferric iron (Fe^3+^). The ferric iron of MetHb is unable to bind oxygen (O_2_). Thus, the O_2_ dissociation curve is left-shifted, making it more difficult to release O_2_ and to provide proper tissue oxygenation. The formation of MetHb also results in less Hb available for O_2_ binding and transport.

MetHb is continuously produced under physiological conditions in a limited amount due to auto-oxidation, but it is rapidly converted back to Hb primarily by nicotinamide adenine dinucleotide (NADH)-dependent cytochrome-b5 reductase (also known as MetHb reductase) and to a limited degree also by ascorbate and glutathione [13]. COHb is formed when Hb and carbon monoxide (CO) interact; CO binds to heme molecules 240 times more than O_2_. The resulting COHb limits the blood’s O_2_-carrying capacity [14]. In healthy, non-smoking individuals, MetHb is in the range of 0.67 ± 0.33% [15] and COHb in the range of 0.5–1.5% [16].

The aim of our study was to summarize the currently available published study results (case reports and cross sectional studies) on MetHb and COHb in patients with COVID-19.

## 2. Methods

The literature research was performed with MEDLINE (PubMed) and Google Scholar using search terms connected with Boolean operators. Two separate searches were performed for MetHb and COHb to make the literature research process simpler. For case one (MetHb and COVID-19) the keywords “methemoglobinemia”, “methemoglobinemia”, ”methemoglobin”, “methaemoglobin”, “COVID-19” and “COVID19” were used; for case two (COHb and COVID-19) they were “carboxy-hemoglobinemia”, “carboxyhaemoglobinaemia”, ”carboxyhemoglobin”, “carboxyhaemo-globin”, “COVID-19” and “COVID19”. Relevant studies published in 2020 were searched. In addition, the reference lists of relevant published articles were also manually searched for additional articles.

The result of the literature research was screened for articles (studies or case reports) that report measurement of MetHb or COHb in COVID-19 patients. The exclusion criteria were articles not reporting MetHb or COHb measurements or articles published in non-academic journals, reviews, dissertations or conference abstracts. Articles published on preprint servers were included.

## 3. Results

### 3.1. Literature Search Results

**Figure 1:**
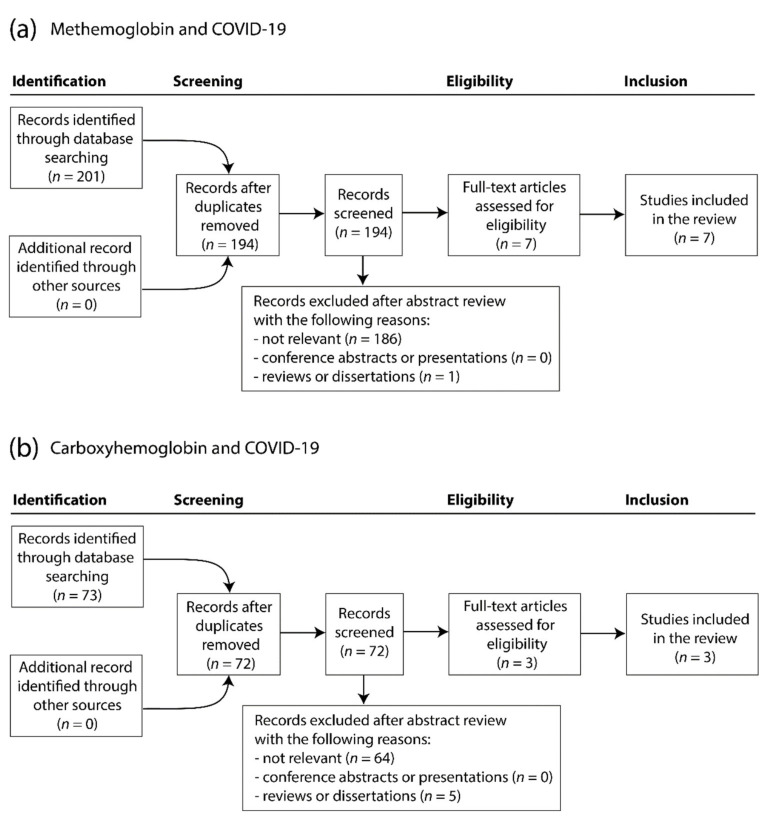
Flow diagram of the literature search process according to the PRISMA criteria. PRISMA: Preferred Reporting Items for Systematic Reviews and Meta-Analyses.

For the case of MetHb and COVID-19, a total of 201 articles were initially retrieved from PubMed (*n* = 6) and Google Scholar (*n* = 196) using the literature search strategy mentioned above. Based on the inclusion and exclusion criteria, seven duplicates were removed and 187 articles were excluded after abstract review. After the full set of remaining articles was screened, seven reports were finally included in the present study [17,18,19,20,21,22,23]. The study selection process is shown in Figure 1a.

For the case of COHb and COVID-19, 73 articles were retrieved from PubMed (*n* = 1) and Google Scholar (*n* = 72), one duplicate was removed and 68 articles were excluded after abstract review, leading to three articles that were finally included in the present study [20,24,25]. The study selection process is shown in Figure 1b.

### 3.2. Characteristics of the Included Studies

From the seven studies included in this review for the case of MetHb and COVID-19, two were cross-sectional studies [19,21] and five case reports [17,18,20,22,23]. The case descriptions reported findings of one single patient [17,20,22,23] or three patients [18]. Five studies were published in journals [17,18,20,21,22] and two on the preprint server medRxiv (medrxiv.org) [19,23].

For the case of the three studies concerning COHb and COVID-19, two were cross sectional studies [24,25] and one a case report [20]. Two of the studies were published in journals [20,25] and one on the preprint server Research Square (researchsquare.com) [24].

### 3.3. Summary of the Studies: MetHb and COVID-19

**Figure 2:**
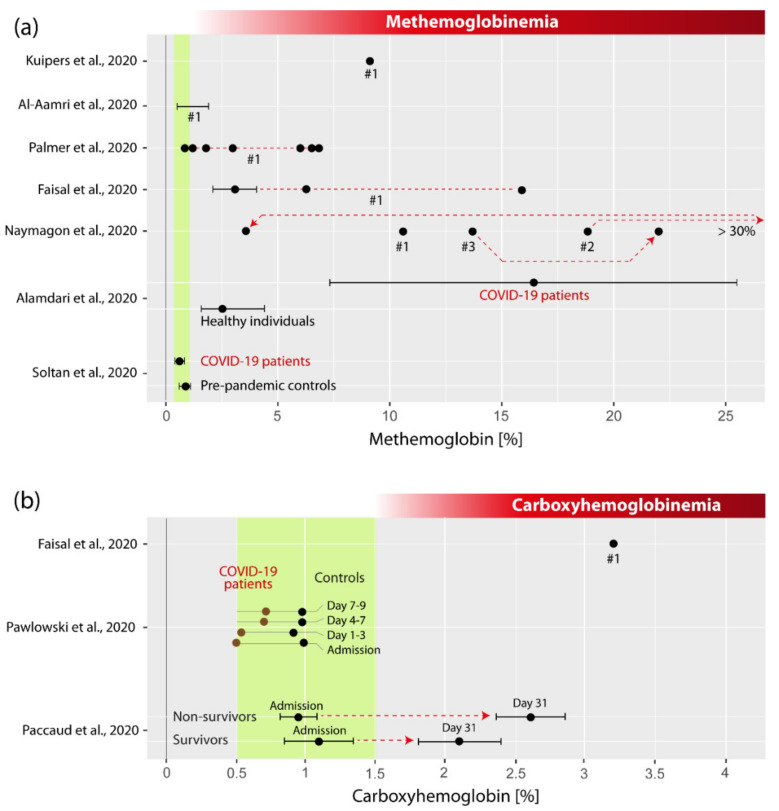
Visualization of (**a**) methemoglobin (MetHb) and (**b**) carboxyhemoglobin (COHb) values of COVID-19 patients reported in the literature. The green shaded areas refer to the reference ranges for healthy individuals in case of MetHb (0.67 ± 0.33% for healthy non-smokers [15]) and COHb (0.5–1.5% for healthy non-smokers [16]). Different subjects reported in the case reports are marked with “#”, and red lines with arrows indicate the development of subject-specific values during the course of the disease.

Kuipers et al. reported the case of a 56-year-old Afro-Caribbean man with a medical history of type 2 diabetes presenting to the emergency department with myalgia, dry cough and a peripheral O_2_ saturation (SpO_2_) of 94%, but not fever. COVID-19 was confirmed by a positive SARS-CoV-2 PCR test result. Bilateral ground glass opacities of the chest were detected by CT. In the following days, an increasing need for O_2_ administration was noted, SpO_2_ dropped to 83% and mechanical ventilation was initiated as well as treatment with chloroquine. Twelve hours later, hemolysis and an abnormally high level of MetHb (9.1%) were noted (Figure 2a). The patient was then treated with three units of packed red blood cells in the following 48 h and ascorbic acid (vitamin C) intravenously four times a day for two days, resulting in a normalization of MetHb levels within six days. A suspected glucose-6-phospate dehydrogenase (G6PD) deficiency was confirmed by genetic analysis.

Al-Aamri described a case of a 10–15-year-old Saudi girl (the exact age was apparently unknown) who was admitted to the hospital with a clinical presentation similar to Kawasaki disease shock syndrome, which she developed 22 days after a routine SARS-CoV-2 test that turned out to be positive. Her condition deteriorated in the following days while receiving multiple medications (Azithromycin, Favipiravir, Methylprednisolone, Enoxaparin, intravenous immunoglobulin, Aspirin, Tocilizumab, Norepinephrine, Epinephrine, Furosemide, Milrinon, insulin, blood transfusion, fresh frozen plasma, vitamin K and ascorbic acid), finally leading to her death at day 33. Intubation was performed and ventilation was started on day 29. The report mentioned that MetHb was 0.5–1.9% (min, max), implying that multiple MetHb measurements were made (Figure 2a). Exactly when the measurements were performed during the disease course was not mentioned. Genetic analysis confirmed a G6PD deficiency.

**Figure 3:**
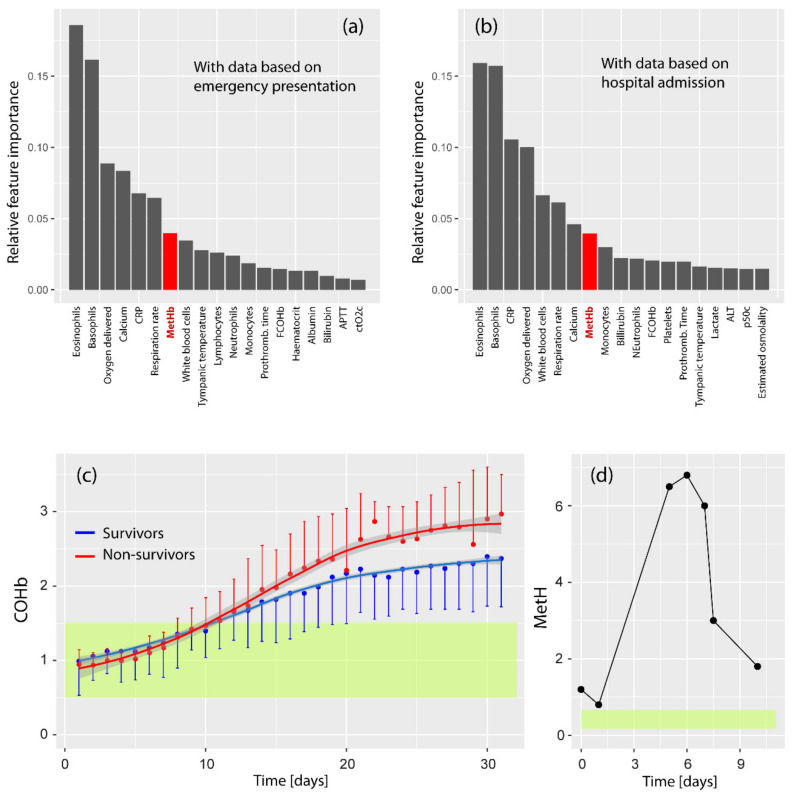
(**a**,**b**) Relative feature importance in predicting COVID-19 according to the study of Soltan et al. [19]. Two models were built, one with data based on emergency presentation (**a**) and one with data based on hospital admission. MetHb was a relevant parameter in both models (indicated in red). (**c**) COVID-19 non-survivors (*n* = 22) show a steeper and higher increase of COHb during the curse of the disease compared to COVID-19 survivors (*n* = 41), according to the study of Paccaudi et al. [24]. The two COHb time-series are statistically significantly different. The yellow bar refers to the COHb reference ranges for healthy non-smokers [16]. (**d**) Time-course of MetHb of a COVID-19 patient reported by Palmer et al. [22]. The yellow bar refers to the MetHb reference range for healthy non-smokers [15]. The figures show data extracted from the original figures of the respective publications.

Palmer et al. reported the case of a 62-year-old Afro-Caribbean man with a medical history of type 2 diabetes and hypertension, presenting at the hospital after five days of fever, dyspnea, vomiting and diarrhea. A chest radiograph showed bilateral infiltrates, a kidney injury was diagnosed and a SARS-CoV-2 test returned positive. On admission, MetHb was 1.2%. The patient was treated with crystalloid fluid, O_2_ therapy, insulin, two blood transfusions, amoxicillin/clavulanic acid, heparin, amlodipine, metformin, and folic acid. The patient was discharged 22 days after admission. On day six, the highest MetHb value was noted (6.8%). A suspected G6PD deficiency was confirmed. The time-course of MetHb during the stay in the hospital is visualized in Figure 3d.

Faisal et al. [20] published a report on a case of a 74-year-old Afro-American man with a medical history of prostate cancer, hypertension and hyperlipidemia that presented to the clinic after seven days with fever, cough and progressively worsening shortness of breath. On admission, the person showed tachypnea, a SpO_2_ of 90% and returned a positive SARS-CoV-2 test result. Chest computed tomography (CT) revealed bilateral perihilar and right lower lobe opacities. After treatment with O_2_ therapy, azithromycin and hydroxychloroquine, his health worsened, and he was intubated and mechanically ventilated. He was then treated with lopinavir-ritonavir, ribavirin, tocilizumab, antibiotics, thiamine, hydrocortisone, ascorbic acid and norepinephrine. On day 15, hypoxia was noted (SpO_2_: 80–90%), but arterial blood gas analysis showed an arterial Hb saturation (SaO_2_) of 100%. MetHb was increased (6.3%). Treatment continued with intravenous ascorbic acid, hydroxocobalamin, and intravenous methylene blue. MetHb raised to 15.9%. After further treatment with intravenous methylene blue and red blood cell transfusion MetHb declined to 2–4%. The patient slowly recovered and was discharged on day 31 after admission. No genetic testing of a G6PD deficiency was performed.

Naymagon et al. [18] reported three cases of COVID-19 patients with MetHb measurements. The first case was a 50-year-old SARS-CoV-2 positive man with no medical history presenting with acute hypoxic respiratory failure in the clinic. He received hydroxychloroquine, azithromycin and ceftriaxone, and mechanical ventilation. He showed persistently low SpO_2_ values despite being mechanically ventilated. MetHb values increased steadily, peaking at 10.6% on day six (Figure 2a). Treatments with methylene blue and ascorbic acid normalized MetHb and his clinical status improved. A genetic analysis of G6PC deficiency was not done. The second case was a 52-year-old SARS-CoV-2 positive morbidly obese man with diabetes mellitus, admitted to the hospital due to acute hypoxic respiratory failure. He received hydroxychloroquine, azithromycin, cefepime, vancomycin, and apixaban, and was mechanically ventilated. A persistently low SpO_2_ was recognized and testing for MetHb resulted in a value of 22% (Figure 2a). He received methylene blue and ascorbic acid, but his MetHb increased to >30%. After receiving red blood cell transfusion, MetHb decreased to 2.9%. No genetic analysis of G6PD deficiency was performed. The third case was a 54-year-old SARS-CoV-2 positive man with diabetes mellitus admitted to the hospital due to acute hypoxic respiratory failure. He received azithromycin and hydroxychloroquine and was mechanically ventilated. He demonstrated persistent hypoxia and a MetHb of 13.7%. After receiving methylene blue, MetHb did not improve and increased to 18.8%. Shortly after, the patient died. Genetic testing revealed G6PD deficiency.

Alamdari et al. [21] investigated MetHb levels in 25 healthy individuals and 25 COVID-19 patients from Iran. Subjects with G6PD deficiency were excluded from the study. Patients showed a statistically significantly higher MetHb concentration in their blood compared to healthy controls (16.4 ± 9.1% vs. 2.5 ± 0.9%). According to the five cases reported in detail in this publication, the medical standard treatment included azithromycin and hydroxychloroquine. To treat the elevated MetHb, methylene blue, ascorbic acid and N-acetyl cysteine were administered. The authors concluded that it is crucial to treat the elevated MetHb in critically ill COVID-19 patients. Soltan et al. [19] used an artificial intelligence method and data from 115,394 emergency presentations and 72,310 admissions to a large UK teaching hospital group to predict COVID-19 cases. The data used were routinely collected data typically available within one hour during emergency presentations and admissions to hospital. Data from COVID-19 patients (*n* = 534) and pre-pandemic controls (*n* = 114,957) were included in the final analysis. Interestingly, while MetHb was a relevant parameter to be included in the models (see Figure 3a,b), MetHb was similar in the COVID-19 cohort compared to pre-pandemic controls (0.62% (0.4–0.8%) vs. 0.88% (0.6–1.1%)).

Figure 2a visualizes the summarized MetHb values reported by the seven publications.

### 3.4. Summary of the Studies: COHb and COVID-19

In their case report on a 74-year-old Afro-American man with COVID-19 already detailed in Section 3.2, Faisal et al. [20] described that, when the patient showed hypoxia and a MetHb concentration of 6.3%, COHb was 3.2%, i.e., above the normal reference range. After the patient was treated with intravenous ascorbic acid, hydroxocobalamin and methylene blue, COHb normalized (value not given in the report), while MetHb continued to rise.

Pawlowski et al. [25] found that, when comparing 246 SARS-CoV-2 PCR-positive patients to propensity-matched 2460 SARS-CoV-2 PCR-negative patients, COHb at clinical presentation was actually slightly, but significantly, higher in the SARS-CoV-2 PCR-negative population (0.99 vs. 0.57%) (Figure 2b). The same trend was seen until seven to nine days after admission.

Paccaudi et al. [24] analyzed the data of 63 patients admitted in the hospital for severe COVID-19 and found that, while COHb was not different for survivors in comparison to non-survivors (1.10 ± 0.50% vs. 0.95 ± 0.24%) (Figure 2b), COHb increased to statistically significantly higher values during the course of the hospital stay in non-survivors compared to survivors (see Figure 3c), leading the authors to conclude that a greater increase in COHb over time seems to represent a relevant marker of COVID-19 severity and seems to play a role in the determination of the survival probability of a COVID-19 infection.

Figure 2b visualizes the MetHb values reported by the seven publications summarized.

## 4. Discussions

### 4.1. Is MetHb Increased in COVID-19 Patients?

According to the case reports of Kuipers et al. [17], Al-Aamri et al. [23], Palmer et al. [22], Faisal et al. [20], and Naymagon et al. [18], MetHb values for COVID-19 patients were above the reference range of 0.67 ± 0.33% for healthy non-smokers (Borland et al. 1985) with the highest MetHb value of >30% for a patient reported by Naymagon et al. [18].

In the case reports of Palmer et al. [22], Faisal et al. [20] and Naymagon et al. [18], MetHb increased in the COVID-19 patients during the course of the disease. Data of multiple MetHb measurements during the disease course were published by Palmer et al. [22], showing a rise of MetHb to a peak after a few days and a decline after treatment (Figure 3d).

COVID-19 patients (*n* = 25) were shown to have a higher MetHb compared to healthy individuals (*n* = 25) as demonstrated in a cross-sectional study by Alamdari et al. [21], supporting the findings reported. However, the cross-sectional study of Soltan et al. [19] with a large cohort (534 COVID-19 patients and 114,957 pre-pandemic controls) showed no statistically significant differences in the MetHb values despite the fact that MetHb was an important parameter for the prediction of COVID-19 based on the algorithm the group developed. This apparent discrepancy between the result of Alamdari et al. and Soltan et al. seems to be due to the following reason: the MetHb data used by Soltan et al. stem from the time of emergency presentations and admission to hospital, whereas the MetHb data from Alamdari et al. were collected from the whole time-course of the hospital stay. Since MetHb has been reported to increase during the development of the disease [18,20,22], the results of Soltan et al. are understandable since, during the MetHb sampling time at the beginning of the disease, MetHb is not necessarily increased (at least at the group level).

In conclusion, MetHb seems to be elevated in COVID-19 patients, with a dynamic following the disease progression.

### 4.2. Is COHb Increased in COVID-19 Patients?

A significantly higher COHb value in COVID-19 patients compared to reference value of healthy non-smokers (0.5–1.5% for healthy non-smokers [16]) was found in two of the three available reports on COHb and COVID-19 published so far. In the case report of Faisal et al. [20], COHb was 3.2% during the disease course. According to the study of Paccaud et al. [24], COHb rose above the reference range on about the 10th day after hospitalization. Interestingly, this study also clearly demonstrated that at admission, COHb was in the normal range for the COVID-19 patients, while COHb was statistically significantly more elevated on the 31st day of the hospital stay in COVID-19 patients compared to patients suffering from other illnesses. These results are in line with the results of Pawloski et al. [25], who reported that their COVID-19 cohort did not show COHb values above the reference range during the time-span investigated (admission until day seven to nine of the stay). Since Paccaud et al. found that it needs about 10 days until COHb is above the reference range, the too short time-span used by Pawloski et al. to compare COHb values from COVID-19 patients and controls might explain the apparent discrepancy.

In conclusion, COHb can be elevated in COVID-19 patients, especially from about two weeks after onset of the disease. The magnitude of COHb elevation seems to be correlated with the survival probability of the COVID-19 patients.

### 4.3. Possible Reasons for Methemoglobinemia in COVID-19 Patients

There are several factors relevant to explain why methemoglobinemia and carboxyhemoglobinemia can be present in COVID-19 patients. The SARS-CoV-2 infection, the individual constitution and the medical treatment seem to be the major ones.

It is well known that several medical drugs can increase MetHb concentration in the blood as a side-effect [26,27]. A recent review reports the early recognition, pathophysiology, and management of methemoglobinemia in the intensive care unit [28]. Chloroquine is such a drug for which reports were published regarding induced methemoglobinemia due to its intake [29,30,31]. Both chloroquine and hydroxychloroquine (a derivative of chloroquine) are currently used to treat COVID-19, while debate is ongoing about their effectiveness and safety [32,33,34,35,36,37,38,39]. Moderately certain evidence suggests that hydroxychloroquine lacks efficacy in reducing short-term mortality in patients hospitalized with COVID-19 or at risk of hospitalization in outpatients with COVID-19.

A G6PD deficiency can enhance the probability of methemoglobinemia induced by oxidizing drugs, such as hydroxychloroquine [40]. However, a very recent experimental animal study suggests that short-course high doses of hydroxychloroquine do not induce methemoglobinemia or clinically significant hemolytic anemia or organ damage in a murine model of G6PD deficiency [41]. Clinically, chronic hemolytic anemia associated with G6PD deficiency is rare [42]. So far, no evidence of hemolysis was observed in patients with G6PD deficiency when exposed to low doses of hydroxychloroquine [43]. The first case of severe hemolytic crisis was found in a seriously ill COVID-19 patient with G6PD deficiency following treatment with high doses of hydroxychloroquine [44]. Several other cases have subsequently been reported by others [45,46,47]. Nevertheless, Mastroianni et al. [47] and Afra [48], concerning hydroxychloroquine use in G6PD-deficient patients, have indicated that it is difficult to assess the relationship between hydroxychloroquine and hemolysis in COVID-19 patients. With the global spread of COVID-19, especially in regions with a high prevalence of G6PD deficiency, these cases should alert physicians to the possible correlation between G6PD-deficiency and hydroxychloroquine treatment.

In the reports on MetHb and COVID-19 summarized in Section 3.2, the use of chloroquine or hydroxychloroquine is described. In the case reported by Kuipers et al. [17], the subject received chloroquine and had a G6PD deficiency; hydroxychloroquine was administered to the subject reported by Faisal et al. [20] (the G6PD status was not reported); and in the three cases reported by Naymagon et al. [18] all received hydroxychloroquine, while one subject was tested for G6PD deficiency and was positive. In the cases reported by Al-Aamri et al. [23] and Palmer et al. [22], the patients did not receive chloroquine and hydroxychloroquine, but the patient described by Al-Aamri et al. had a G6PD deficiency. A G6PD deficiency is not only relevant with respect to the reaction to antiviral oxidizing drugs, but also for the effects of the drug methylene blue administered to treat the methemoglobinemia. Methylene blue may be ineffective in patients with a G6PD deficiency since they lack sufficient reduced nicotinamide adenine dinucleotide phosphate (NADPH) to reduce methylene blue to leuko-methylene [49,50].

Despite the obvious effect of oxidizing drugs on the formation of MetHb, it can also be formed as a byproduct of a physiological reaction in the form of an adaptive increased nitric oxide (NO) signaling due to an acute anemia [51]. Anemia can be associated with an infection and/or a systemic inflammatory reaction, termed “anemia of inflammation”, as part of the physiological reaction to the disease [52,53]. According to a study by Bellmann-Weiler et al. [54] on 259 hospitalized patients with COVID-19, 24.7% were anemic on admission, with the majority suffering from anemia of inflammation (68.8%). During hospitalization, the percentage of patients with anemia increased (around 68.8% at day 7). A significantly higher mortality during hospitalization was also found in those with anemia upon admission. Anemia is associated with an increased NO expression, leading to vasodilation and thus preventing tissue hypoxia, but also causing increased NO-based oxidation of Hb to MetHb [51]. Interestingly, anemia, and drops in total Hb (tHb), have been reported in COVID-19 patients [22,55,56].

An anticorrelated development of tHb levels and MetHb levels in a COVID-19 patient has been reported [22], in line with the pathway described by Hare et al. [51] of an NO-induced MetHb increase caused by anemia. The role of MetHb as a marker of anemic stress has been also been validated in a study investigating MetHb changes in patients undergoing heart surgery [57]. While “anemia of inflammation” is associated with methemoglobinemia, iron-deficient anemia seems to be a further risk factor for acquired methemoglobinemia by enhancing red blood cell oxidative stress [58]. The occurrence of methemoglobinemia due to a viral infection has been reported for several types of infection [59,60,61]. For example, the activity of MetHb reductase has been shown to be negatively affected by infections with *Plasmodium yoelii nigeriensis* [62,63] or *Plasmodium knowlesi* [61].

The fact that the severity of the methemoglobinemia observed in COVID-19 patients was dependent on the subject firstly reflects the different disease severities of the patients but is also most probably due to the physiological constitution of the subjects before the disease, related to their medical history, their overall fitness and age. The age factor seems to be of particular interest since, for example, erythrocytes from elderly humans are more easily affected by oxidative stress, facilitating the formation of MetHb [64].

MetHb can have proinflammatory properties. For example, it activates the NF-κB pathway in endothelial cells associated with chemokine (IL-8) and cytokine (IL-6) production [65]. The activation of the NF-κB and MAPK pathways with subsequent release of the chemokines IL-8 and the chemokine monocyte chemoattractant protein-1 (MCP-1) has also been observed in endothelial cells exposed to MetHb [66]. This underlines that an elevation of MetHb in the blood has an effect on cytokine/chemokine production—a fact that might be of particular relevance for COVID-19 since a “cytokine storm” has been observed in severe courses of the disease [67,68,69,70]. At the same time, it must also be borne in mind that hypoxia also causes the production of cytokines and cytokines, like IL-8 and IL-6 [71,72].

### 4.4. Possible Reasons for Carboxyhemoglobinemia in COVID-19 Patients

Since the blood COHb concentration reflects the balance between endogenous CO production and CO elimination, carboxyhemoglobinemia in COVID-19 patients could indicate an increased endogenous CO production and/or a decreased CO elimination ability.

Endogenous CO production is mainly due to the inducible heme oxygenase (HO-1) enzyme, which catalyzes the heme moiety of Hb to biliverdin and liberates CO during this process. CO can then react with Hb, leading to the formation of COHb. HO-1 is upregulated in case of oxidative stress and inflammation which leads to increased COHb production [73,74]. Hemolytic anemia facilitates the production process of COHb so that an increased COHb blood level can be seen as a manifestation of hemolytic anemia [75]. Since anemia and hemolysis possibly occur during the course of disease in COVID-19 patients [17,18,22,54], hemolytic anemia may also be responsible for their COHb elevation. Because intracellular NADPH depletion and consecutive oxidative stress with damaged erythrocytes (hemolysis) is typical for G6PD deficiency, it is not surprising that G6PD deficiency in COVID-19 patients seems to be associated with elevated MetHb and COHb levels [20].

A decreased CO elimination occurs when respiration is impaired. As COVID-19 patients are characterized by respiratory impairment, increased COHb levels can be explained by reduced CO elimination and thus a higher probability of COHb formation. Mechanical ventilation may also be relevant since, for example, an increase in the inspired O_2_ fraction leads to an increase in exhaled CO concentration [76], possibly leading to a reduced COHb production.

Interestingly, while elevated COHb levels seem to be correlated with COVID-19 severity, intensive care mortality from other causes was found to be associated with too low [12,77] and both too low or too high COHb values [11], indicating the existence of an optimal COHb level for optimal physiological functioning [12]. HO-1 upregulation, associated with elevations of COHb, has immunomodulatory and anti-inflammatory effects [78]. Inflammation changes COHb levels in the blood in a complex time-dependent manner as demonstrated by experimental endotoxemia in humans [79], highlighting the non-linear relationship between inflammation, disease severity and COHb levels.

### 4.5. Methemoglobinemia and Carboxyhemoglobinemia in COVID-19 Patients: Consequences for Patient Monitoring and Treatment

Methemoglobinemia and carboxyhemoglobinemia seem to play a role in the pathophysiology of COVID-19, especially in more severe cases of the disease.

While the ability to determine MetHb and COHb levels is normally routinely available in clinical settings via blood gas analysis, there are only a few commercial monitoring devices that enable continuous non-invasive measurement of MetHb and COHb values, mainly the fingertip pulse CO-oximeters by Masimo and Nonin. These devices do not normally feature in standard clinical equipment. This is unfortunate since continuous monitoring of tHb, MetHb and COHb levels could be helpful in guiding the treatment and monitoring of COVID-19 disease progression.

From the reports discussed in Section 3.2 and Section 3.3 it is clear that the determination of MetHb and COHb is especially warranted when patients are treated with oxidizing drugs such as chloroquine and hydroxychloroquine. This is even more important when the patients have a confirmed G6PD deficiency.

For the interpretation of COHb values, the smoking status of the patient needs to be considered as smokers have a higher COHb concentration in the blood than non-smokers (2.7 ± 2.6% [80], 3.26 ± 2.2% [15], 5.12 ± 2.25% [81], 2.1 ± 1.02% [82]).

**Figure 4:**
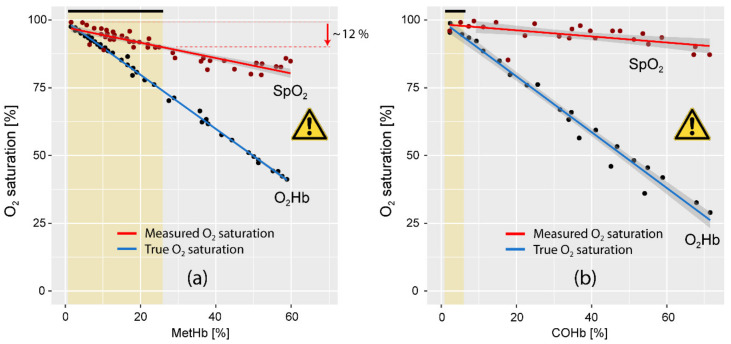
The impact of MetHb and COHb on SpO_2_ measured with pulse oximetry. (**a**) Blood MetHb vs. SpO_2_ and blood Hb O_2_ saturation (O_2_Hb). Data extracted from Barker et al. [86]. (**b**) Blood COHb vs. SpO_2_ and blood O_2_Hb saturation. Data extracted from Barker et al. [87]. In both studies, measurements were made on dogs; the inspired O_2_ fraction (FIO_2_) was 1, SpO_2_ was measured on the tongue with a pulse oximeter (Nellcor N-100, USA) and COHb, MetHb and O_2_Hb saturation with a CO-oximeter (IL-282, Instrumentation Laboratories, Bedford, MA, USA). The figures show data extracted from the original figures of the respective publications.

Knowledge of MetHb and COHb levels in the blood of COVID-19 patients is also relevant to prevent misinterpretations of arterial oxygen saturation values measured with fingertip pulse oximetry (SpO_2_). This is because MetHb and COHb interfere with the measurement of SpO_2_. An overestimation of the true arterial oxygenation (SaO_2_) can occur. In case of a decrease in SaO_2_ and an increase in MetHb or COHb, SpO_2_ will diverge more from SaO_2_ the higher the MetHb and COHb concentration (see Figure 4). For example, assuming a MetHb concentration of around 25% (corresponding to the upper end of the confidence interval of MetHb values in COVID-19 patients reported by Alamdari et al. [21]) and an assumed decrease of SaO_2_ to 75%, the SpO_2_ measurements would indicate a falsely too high SpO_2_ of about 88%. Measurement with pulse CO-oximetry instead of pulse oximetry would circumvent this problem since pulse CO-oximetry is able to non-invasively measure MetHb, COHb, tHb, and the correct SpO_2_. [83,84,85].

Methemoglobinemia and carboxyhemoglobinemia cause a shift in the Hb dissociation curve to the left, leading to a reduced ability of O_2_ to be released from Hb, which can result in hypoxia. Methemoglobinemia and carboxyhemoglobinemia need to be monitored and treated, therefore. Therapies of methemoglobinemia in COVID-19 patients have been performed with methylene blue and, in some cases, combined with blood transfusions [18,20,21].

The fact that COVID-19 patients could show hypoxemia without having dyspnea, i.e., silent hypoxia or so-called “happy” hypoxia [88,89,90], seems to be primarily due to (i) a blunted response of the respiratory control system to hypoxia which is prevalent in older subject and those with diabetes, (ii) changes in arterial CO_2_ levels, (iii) temperature-induced shifts in the O_2_ dissociation curve, and (iv) the inaccuracy of pulse oximeters at low SpO_2_ values, as highlighted by Tobin et al. [89,91].

For hospitals it might be also relevant to re-evaluate their water disinfection procedure since the use of a hydrogen peroxide/silver ion preparation for treating the water supplied in the hospital caused elevated levels of MetHb in the severely ill patients (treated with daily hemodialysis/hemodiafiltration) drinking this water [92]. When blood transfusions are given to COVID-19 patients, it should be also considered that the MetHb content of the banked blood increases over time [93] and that banked blood from smoking donors can have a relatively high COHb concentration [94], representing a possible risk for critically ill patients. It makes sense therefore to test the banked blood for MetHb and COHb concentration levels before administering it to patients, especially in case of COVID-19.

## 5. Summary and Conclusions

We identified and analyzed nine studies that reported information about MetHb and COHb values of COVID-19 patients. 86% (6/7) studies reported an increased MetHb concentration above the normal reference range, and one study did not find such an increase. Regarding COHb, 67% (2/3) studies reported an increase, and one found no elevated COHb. As highlighted by the observations of Palmer et al. [22] and Paccaud et al. [24], the time of MetHb and COHb measurement during the course of the disease is critical, since MetHb and COHb seem to generally increase in hospitalized COVID-19 patients. The type of medical treatment is also of importance since some drugs currently used to treat COVID-19 have a high oxidizing potential provoking methemoglobinemia and carboxyhemoglobinemia.

Monitoring of MetHb and COHb values routinely, and if possible, continuously with non-invasive pulse CO-oximeters, seems to be warranted for optimal COVID-19 disease monitoring. The tHb, MetHb and COHb concentration in the blood is also relevant for a correct interpretation of SpO_2_ values measured with fingertip pulse oximetry.

Future studies should investigate in detail the link between MetHb/COHb levels in COVID-19 patients and the clinical outcome. As both values were found to be independently related to mortality in critically ill patients [77], the assessment of both parameters is necessary.

In conclusion, MetHb and COHb seem to be elevated in COVID-19 patients, with a dynamic following the disease progression. MetHb and COHb measurements should be regularly assessed in COVID-19 patients, for example with blood gas analysis (which includes a CO-oximetry module), conventional CO-oximeters or by continuous CO-oximetry.
